# Gene Polymorphism of Matrix Metalloproteinase 9 in Asthenozoospermic Male Subjects

**DOI:** 10.22074/ijfs.2018.5038

**Published:** 2017-10-14

**Authors:** Sina Mohagheghi, Iraj Khodadadi, Manoochehr Karami, Iraj Amiri, Heidar Tavilani

**Affiliations:** 1. Student Research Center, Hamadan University of Medical Sciences, Hamadan, Iran; 2. Department of Biochemistry, Faculty of Medicine, Hamadan University of Medical Sciences, Hamadan, Iran; 3. Modeling of Non-Communicable Diseases Research Center, Department of Biostatistics and Epidemiology, School of Public Health, Hamadan University of Medical Sciences, Hamadan, Iran; 4. Endometrium and Endometriosis Research Center, Hamadan University of Medical Sciences, Hamadan, Iran; 5. Urology and Nephrology Research Center, Hamadan University of Medical Sciences, Hamadan, Iran

**Keywords:** Infertility, Matrix Metalloproteinase, Polymorphism, Semen, Single Nucleotide

## Abstract

**Background:**

Matrix metalloproteinase (MMPs) play important roles in the structural and functional properties of reproductive organs. The aim of this study is to determine the prevalence of C-1562T *MMP-9* (rs3918242) gene polymorphism in fertile and infertile men. In addition, we aim to determine the association between C-1562T *MMP-9* and G-1575A *MMP-2* gene polymorphisms.

**Materials and Methods:**

A total of 400 subjects, including 200 fertile and 200 infertile men, were recruited for this casecontrol study. The allele frequencies and genotype distributions of single nucleotide polymorphism in the promoter regions of *MMP-9* (C-1562T) were determined using polymerase chain reaction-restriction fragment length polymorphism (PCR-RFLP) analysis. The chi-square (χ2) test was used to assess the distribution of genotype frequencies.

**Results:**

There were no significant differences found in the genotype distributions or allele frequencies between fertile and infertile men for the C-1562T *MMP-9* gene polymorphism. The percent of immotile sperm in infertile men with the CC and CT genotypes of C-1562T *MMP-9* gene polymorphism significantly differed compared with that of subjects with the TT genotype. The frequency of CC/GA-combined genotypes of C-1562T *MMP-9* and G-1575A *MMP-2* gene polymorphisms significantly differed in fertile and infertile men (P=0.031).

**Conclusion:**

Our results suggest that genetic polymorphisms in MMP may impact male fertility.

## Introduction

Infertility is a clinical problem that affects married couples. Nearly 20% of couples experience infertility and 50% of these cases are related to male reproductive disorder (1). Male infertility is influenced by many environmental and genetic factors (2,3). Reproductive organs undergo major alterations in their structural and functional properties throughout adult life. These changes involve remodeling of connective tissues. Matrix metalloproteinases (MMPs), due to their specific features, play important roles in these modifications (4). >Mammals have 28 types of MMP (5). These enzymes require zinc for their activities and are involved in the degradation of both the extracellular matrix and basement membrane. MMPs and their tissue inhibitors (TIMPs) participate in a number of physiological processes, such as ovulation and fertilization. These enzymes have been frequently studied in the female reproductive system, but not extensively examined in terms of male fertility. One study showed that the MMP profile between normal and abnormal sperm samples differed and suggested that sperm abnormality might occur because of the presence or absence of a specific MMP (6). Accumulating evidence has shown that MMPs impact male fertility for three reasons. First, these enzymes play an important role in the development of reproductive organs (7). Second, MMPs are required for spermatogenesis (1). Third, the breakdown of physical barriers between egg and sperm and the contact of sperm with the egg surface, along with certain specific features of MMPs, suggest that they may be involved in fertilization (8,9). 

Gelatinases, a subtype of MMPs (including MMP-2 and-9), are usually involved in the degradation of collagen type IV and gelatine (10,11). MMP-2 (collagenase A with a molecular weight of 72 kDa) and MMP-9 (collagenase B with molecular weight of 92 kDa) present in the acrosome and tail of the sperm, respectively (12). Single-nucleotide polymorphisms (SNPs) in the promoter region of *MMP* genes may influence their expression and thus alter their enzyme activities. Several polymorphisms, such as G-1575A of *MMP-2* and C-1562T of *MMP-9*, have been identified in the promoter region of *MMP-2* (ID 4313) and *MMP-9* (ID 4318) genes, which affect their expression (13). In our previous report, we have determined the G-1575A genetic polymorphism of *MMP-2* in fertile and infertile men. Our result showed that the frequencies of GA genotype in fertile and infertile men significantly differed (14). The C-1562T gene polymorphism in the *MMP-9* gene is located at position -1562 of the promoter site, where a transition occurs between C and T. This site is relative to the transcription start site. The C allele results in lower promoter activity than the T allele due to its lower affinity to a nuclear protein (13). 

Considering the role of *MMP-9* in both remodeling and destruction of the extracellular matrix, genetic variations may affect the transcription of the gene and activity of this enzyme, resulting in a propensity towards male infertility. To the best of our knowledge, the prevalence of C-1562T *MMP-9* gene polymorphisms as well as the association of C-1562T *MMP-9* and G-1575A of *MMP-2* gene polymorphisms has not been previously investigated in male fertility. Therefore, the aim of this study was to determine the prevalence of C-1562T *MMP-9* (rs3918242) gene polymorphism in fertile and infertile men. In addition, the association between C-1562T of *MMP-9* and G-1575A of *MMP-2* gene polymorphism in fertile and infertile men was determined. 

## Materials and Methods

A total of 400 subjects (200 fertile and 200 infertile men) participated in this study. A literature review showed no previously published study on the C-1562T *MMP-9* gene polymorphism in male fertility; therefore, we determined the sample size based on the predominant genotype frequency of the C-1562T *MMP-9* polymorphism in the population. Based on the average frequency of the predominant genotype in the study population (85%), the minimum difference in dominant genotype frequency between two groups was set at 10%, with a type I error set at α=0.05 and type 2 error considered to be β=20%. 

The fertile men were staff members of the Hamadan University of Medical Sciences who voluntarily participated in this study. Fertile individuals had no specific diseases. Infertile men were randomly selected from patients admitted to the Fatemieh Fertility Clinic at Hamadan University of Medical Sciences and all had abnormal spermogram results according to World Health Organization laboratory guidelines (2010). In this case-control study, the fertile men had children born within the past 5 years. Patients had idiopathic infertility (failure to achieve a clinical pregnancy after 12 months or more of regular unprotected intercourse). We excluded all cases with specific reasons for their disease, such as varicoceles and abnormal karyotype (15) from the study. Subjects from two fertile and infertile groups were matched for age (29-42 years) to achieve no significant difference between the two groups. All participants provided written informed consent and the Research Ethics Committee of Hamadan University of Medical Sciences approved this study. 

## Semen analysis

We collected and examined semen samples from 200 infertile men according to the World Health Organization (2010) laboratory guidelines (16). The ejaculates were collected into sterile containers during masturbation after at least 72 hours of sexual abstinence and allowed to liquefy at room temperature. Semen suspensions were analyzed for sperm concentration, linear progressive movement (motility), and morphology. The infertile individuals were divided into asthenozoospermic and teratoastheozoospermic subjects. Criteria for asthenozoospermia was defined by progressive motility<32%, sperm concentration ≥20×10^6^/ml, and normal morphology ≥15%. Criteria for teratoastheozoospermia was defined by progressive motility <32%, sperm concentration ≥20×10^6^/ml, and normal morphology<14%.

## DNA extraction

We collected 3 ml whole blood into EDTA coated tubes. Genomic DNA was extracted using the ethanol-chloroform extraction method (17). DNA concentration was determined by spectrophotometry at 260 nm. The *MMP-9* nucleotide polymorphism at position -1562 was determined by polymerase chain reaction (PCR) combined with the restriction fragment length polymorphism (RFLP) method using forward, (5ˊ-GCCTGGCACATAGTAGGCCC-3ˊ) and reverse, (5ˊ-CTTCCTAGCCAGCCGGCATC-3ˊ) primers (18). For PCR of each sample, a premix PCR kit (Bioneer, Korea) was used. For the PCR cycle, after DNA denaturation at 94°C for 5 minutes, the reaction mixture was subjected to 30 cycles at 94°C for 45 seconds, 55°C for 45 seconds, and 72°C for 45 seconds with a final extension time of 5 minutes. Electrophoresis was performed with Syber Safe staining on 2% agarose gel to confirm the 435 bp size of the PCR product. 

## Genotyping

**Fig.1:**
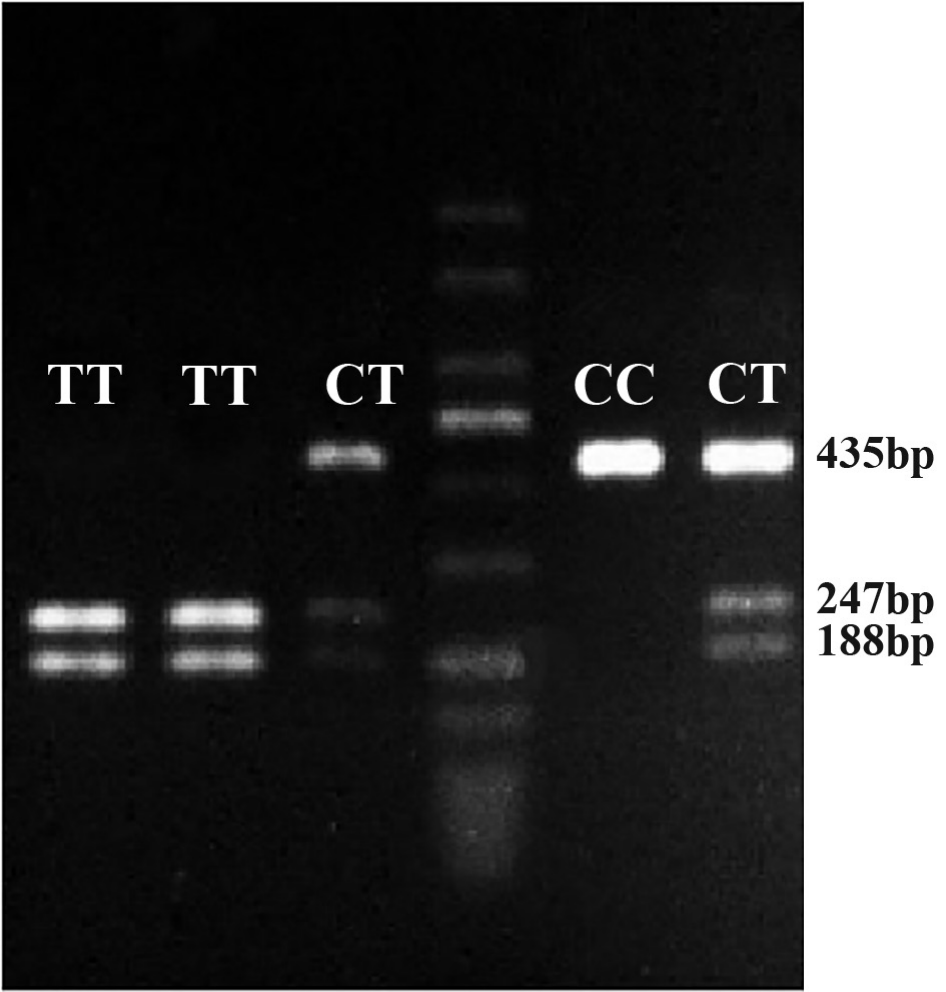
The polymerase chain reaction-based SphI restriction fragment length polymorphism (PCR-RFLP) products for C-1562T *MMP-9* gene polymorphism on agarose 2%. The PCR products were digested by sphI restriction endonuclease. The CC genotype produced a 435 bp,TT genotype 247bp and 188 bp, and CT genotype 435 bp, 247 bp and 188 bp PCR product.

To analyze the presence of polymorphism, PCR products were digested using the sphI restriction enzyme. Amplified PCR products (10 µl) were digested in a 30 µl final reaction volume that consisted of 2 µl of 10x Reaction Buffer and 5 units of sphI restriction endonuclease (Fermentas, USA), incubated at 37°C overnight. The enzyme digested products for *MMP-9* gene were analyzed on a 2% agarose gel. Gels pre-stained with Syber Safe (for visualization under a UV light) were run at 96 mV in 1X tris-borate-EDTA (TBE) buffer for 45 minutes. The homozygote CC genotype yielded a 435 bp product, whereas we observed two fragments (247 bp and 188 bp) for the TT homozygote subjects. Heterozygotes with the CT genotype, on the other hand, yielded three PCR product fragments of 435 bp, 247 bp, and 188 bp after *sphI* digestion (Fig .1). DNA sequencing confirmed the genotypes identified by the RFLP method. 

## Statistical analysis

All the statistical analyses were carried out using the Statistical Program of Social Sciences (SPSS) version 16.0 (SPSS Inc., Chicago, IL, USA). Data were presented as mean ± standard deviation and statistical significance was defined as P<0.05. The chi-square (χ2) test was used to assess the distribution of genotype frequencies for deviation from the Hardy-Weinberg equilibrium. We calculated the allele frequencies from the observed numbers of genotypes. Odds ratios (OR) with 95% confidence intervals were calculated as estimates of relative risk for infertility. 

## Results

We examined the association between the C-1562T polymorphism in the *MMP-9* gene and the risk of male infertility. The homozygous CC genotype was detected in 59% of the fertile men compared to 66% of the infertile men. The homozygous TT genotype was detected in 4.5% of the fertile men and 3.5% of the infertile men. The heterozygous CT genotype was detected in 36.5% of fertile men and 30.5% of infertile men. The frequencies of CT and TT genotypes did not show significant differences between fertile (P=0.174) and infertile (P=0.482) men. 

We determined allele frequencies of the C-1562T* MMP-9* gene polymorphism (Table 1). There was no association found between the allele frequencies of the C-1562T *MMP-9* gene polymorphism and the risk of infertility. Table 1 shows the genotype and allele OR of the C-1562T *MMP-9* gene polymorphism in fertile and infertile men. 

Our results indicated that the variant genotype -1562 CC in the *MMP9* gene did not have a significant association with an increased risk of infertility [adjusted OR=1.34, 95% confidence interval (CI): 0.88-2.02 and OR=1.44, 95% CI: 0.52-3.98]. 

**Table 1:** The frequency of C-1562T MMP-9 genotypes, alleles, and odds ratios in fertile and infertile males


C-1562T MMP-9	Infertile men n=200	Fertile men n=200	OR (95% CI)

C/C^a^	132 (66%)	118 (59%)	1.00
C/T	61 (30.5%)	73 (36.5%)	1.34 (0.88-2)
T/T	7 (3.5%)	9 (4.5%)	1.44 (0.52-3.98)
Alleles			
C^a^	325 (81.25%)	309 (77.25%)	1.00
T	75 (18.75%)	91 (22.75%)	1.27 (0.9-1.8)


^a^; Reference group, OR; Odds ratios, and CI; Confidence interval.

The semen profiles of infertile subjects with reference to the MMP-9 genotypes are described (Table 2). The percent of immotile sperm in infertile men with the CC and CT genotypes of C-1562T *MMP-9* gene polymorphism showed a significant difference compared to subjects with the TT genotype. According to the spermiogram, we divided the infertile men into two groups: asthenozoospermia (n=97) and terato-asthenozoospermia (n=103). We determined the genotype distribution of C-1562T *MMP-9* gene polymorphism in the three groups of fertile, asthenozoospermia, and terato-asthenozoospermia (Table 3). We found no statistically significant difference in genotype distribution of the C-1562T polymorphism in these three groups. Since we previously reported the genotype and allele frequencies of the G-1575A *MMP-2* gene polymorphisms in these subjects (14), in the current study, we analyzed the association of the G-1575A *MMP-2* and C-1562T *MMP-9* gene polymorphisms. The results demonstrated that the frequencies of CC/GA-combined genotypes of C- 1562T MMP-9 and G-1575A MMP-2 gene polymorphisms had a significant difference between fertile and infertile men (P=0.032, χ2=4.6, df=1) (Table 4). We also analyzed the synergistic effect of alleles C-1562T MMP-9 and G-1575A MMP-2 gene polymorphisms on male infertility (Table 5). Synergistic analysis of the allele frequencies of G-1575A and C-1562T gene polymorphisms showed that the frequency of individuals with negative T allele (MMP- 9) and positive A allele (MMP-2) between fertile and infertile men significantly differed (P=0.038).

**Table 2:** Semen profiles of infertile men regarding genotype of C-1562T MMP-9 gene polymorphism


C-1562T MMP-9	Motility (%) (mean ± SD)			Sperm count (million/ml)	Normal morphology (%)
	Progressive	Non progressive	Immotile		

C/C (n= 132)	7.24 ± 7.95	17.97 ± 13.05	57.4 ± 25.2	44.17 ± 26.77	19.37 ± 11.05
C/T (n=61)	8.65 ± 8.05	16.32 ± 10.3	55.43 ± 27.39	40.69 ± 31.01	20.53 ± 14.8
T/T (n=7)	7.15 ± 8.72	16.73 ± 23.74	20.85 ± 27.27	35.5 ± 41.49	14.16 ± 13.93
P value	0.312	0.734	0.004	0.630	0.481


**Table 3:** The genotype distribution of C-1562T MMP-9 gene polymorphism in three groups of fertile, teratoasthenozoospermia and asthenozoospermia infertile men


C-1562T MMP-9	Fertile men n=200	Infertile men n=200		P value
		Teratoasthenozoospermia n=103	Asthenozoospermia n=97	

C/C^a^	118 (59%)	68 (66.02%)	64 (65.98%)	
C/T	73 (36.5%)	31 (30.09%)	29 (29.89%)	0.358 (χ^2^=2, df=2)
T/T	9 (4.5%)	4 (3.89%)	4 (4.13%)	0.891 (χ^2^=0.22, df=2)


^a^; Reference group, NA; Not applicable, OR; Odds ratios, CI; Confidence interval, x^2^; Chi-square, and df; Degree of freedom.

**Table 4:** The combined genotype frequencies of G-1575A MMP-2 and C-1562T MMP-9 gene polymorphisms in fertile and infertile men


MMP-9 -1562/MMP-2-1575	Infertile men	Fertile men	OR (95% CI)	P value

CC/GG^a^	81 (40.5%)	57 (28.5%)	1.00	
CC/GA	48 (24%)	59 (29.5%)	1.74 (1-2.9)	0.032 (χ^2^=4.6, df=1)
CC/AA	4 (2%)	2 (1%)	0.7 (0.12-4)	0.702 (χ^2^=0.151, df=1)
CT/GG	37 (18.5%)	45 (22.5%)	1.72 (0.99-3)	0.051 (χ^2^=3.81, df=1)
CT/GA	22 (11%)	27 (13.5%)	1.74 (0.9-3.36)	0.093 (χ^2^=2.78, df=1)
CT/AA	1 (0.5%)	1 (0.5%)	1.42 (0.09-23.2)	0.814 (χ^2^=0.061, df=1)
TT/GG	6 (3%)	5 (2.5%)	1.18 (0.35-4)	0.794 (χ^2^=0.072, df=1)
TT/GA	1 (0.5%)	4 (2%)	5.6 (0.6-52)	0.093 (χ^2^=2.95, df=1)
TT/AA	0	0	NA	NA


^a^; Reference group, OR; Odds ratios, CI; Confidence interval, x^2^; Chi-square, and df; Degree of freedom.

**Table 5:** Synergism of alleles of G-1575A MMP-2 and C-1562T MMP-9 gene polymorphisms in fertile and infertile men


MMP-9 -1562/MMP-2-1575	Infertile men	Fertile men	OR (95% CI)	P value

Both negative MMP9 T allele and MMP-2 A allele^a^ (CC+GG)	81 (40.5%)	57 (28.5%)	1.00	
Negative MMP9 T allele and positive MMP-2 A allele (CC+AA+GA)	51 (25.5%)	61 (30.5%)	1.7 (1-2.8)	0.038 (χ^2^=4.29, df=1)
Positive MMP9 T allele and negative MMP-2 A allele (TT+CT+GG)	44 (22%)	50 (25%)	1.29 (1-1.7)	0.071 (χ^2^=3.18, df=1)
Both positive MMP9 T allele and MMP-2 A allele (TT+CT+AA+GA)	24 (12%)	32 (16%)	1.9 (1-3.5)	0.045 (χ^2^=4, df=1)


^a^; Reference group, OR; Odds ratios, CI; Confidence interval, x^2^; Chi-square, and df; Degree of freedom.

## Discussion

In this study we determine the prevalence of the C-1562T *MMP-9* gene polymorphism and its association with the G-1575A of MMP-2 gene polymorphism in fertile and infertile men. The study indicated that in the C-1562T *MMP-9* gene polymorphism, the frequencies of CT and TT genotypes did not significantly differ in fertile and infertile men. The risk of infertility in individuals with the CC genotype was 1.3-times more than individuals who carried the CT genotype and 1.4-times more than those with the TT genotype; however, these associations did not reach statistical significance. Based on logistic regression analysis, the T allele could have a protective effect and possibly decrease the risk of male infertility by approximately 1.27 times, however the P value was not statistically significant. The number of participants with the T allele in the fertile and infertile groups was most likely not adequate to reach a statistically significant conclusion. It was reported that the T allele carriers of C-1562T *MMP-9* gene polymorphism had a higher enzyme activity and protein level compared to C allele carriers (19). Since nuclear proteins have a lower affinity to the C allele compared to the T allele, individuals with the C allele have a lower promoter function, and consequently, a lower MMP-9 enzyme activity (13). Therefore, most likely, the T allele increases the expression of *MMP-9* and has a positive effect on male fertility. 

Our results were consistent with the results of a study conducted by Patricia et al. which showed that the TT genotype in the C-1562T *MMP-9* gene polymorphism had a protective effect against the development of lung cancer compared to the reference genotype (20). Here we confirmed that the TT genotype in the C-1562T *MMP-9* gene polymorphism had a protective effect on sperm motility and might indirectly improve male fertility. On the other hand, Wang and Shi (21) showed that East Asian T allele carriers (TT+TC) compared with C allele carriers had a significantly higher risk of coronary artery diseases. These results suggested that this polymorphism could have different effects in different diseases. 

In this study, we divided the infertile men into asthenozoospermic and terato-asthenozoospermic groups according to their semen profiles, and determined the frequencies of the genotype C-1562T *MMP-9* gene polymorphisms. We found that genotype distribution of C-1562T polymorphism in asthenozoospermic, terato-asthenozoospermic, and fertile men did not statistically differ. These results suggested that the C-1562T *MMP-9* gene polymorphisms had no significant effect on the morphology of spermatozoa. In accordance with these observations, semen analysis showed that the genotype frequencies of the C-1562T *MMP-9* gene polymorphisms were not significantly different in terms of morphology. On the other hand, we found a lower percentage of immotile sperm in men who carried the TT genotype compared to the CC and CT genotype carriers of the C-1562T *MMP-9* gene polymorphism. 

Ferrer et al. (12) identified MMP-9 activities in human spermatozoa that were mainly in the mid-piece of the sperm tail. According to previous reports, T allele carriers of the C-1562T *MMP-9* gene polymorphism had higher MMP-9 activity and protein level compared to C allele carriers (19). We could explain the lower percentage of immotile sperm from the TT genotype compared to the CT and CC genotypes. On the other hand, higher MMP-9 activity in the sperm tail of males with the TT genotype might improve sperm cell motility. However, we did not determine the MMP-9 enzyme activity in participants enrolled in this study. In addition, due to ethical issues, we did not have access to the semen of fertile men to strengthen our conclusion. 

*MMP-9* gene polymorphisms could be associated with other *MMP* polymorphisms in the genome. We analyzed the synergism of genotypes and alleles of G-1575A *MMP-2* and C-1562T *MMP-9* gene polymorphisms on male infertility. In our previous report on G-1575A MMP-2 polymorphism, we showed that the frequencies of GA genotype of fertile and infertile men were significantly different; however, the frequency of AA and GG genotypes didn’t show any significant differences. In G-1575A *MMP-2* polymorphism, the risk of infertility in individuals with AA genotype was 2.14-fold more than individuals carrying GA genotype (14). The synergistic analysis of genotypes of C-1562T *MMP-9* and G-1575A *MMP-2* gene polymorphisms showed that the frequency CC/GA-combined genotype was significantly different between fertile and infertile men. It demonstrated a protective effect which could increase male fertility about 1.7 times. 

The synergistic effects of different MMP polymorphisms on male infertility is very complex, and further investigations with larger sample size are needed to clearly delineate the impact of *MMP* polymorphisms on male infertility. On the other hand, synergistic analysis of alleles *G-1575A MMP-2* and *C-1562T MMP-9* gene polymorphisms showed that the frequency of individuals with negative MMP-9 T allele and positive MMP-2 A allele between fertile and infertile men was significantly different. We also found that the frequency of individuals with both positive MMP-9 T allele and MMP-2 A allele was significantly different in the fertile group compare with the infertile individuals. 

A previous study has shown that the systemic lupus erythematous (SLE) patients with A allele (GA+AA genotypes) of G-1575A MMP-2 gene polymorphism have higher levels of MMP-2 activity than the control subjects (22). Two polymorphisms that were examined in this study (*G-1575A MMP-2* and *C-1562T MMP-9*) are located in the promoter regions of their corresponding genes. Promoter regions of *MMP-2* and *MMP-9* contain regulatory elements which are affected by transcription factors. The substitution of T with C in position of -1562 from *MMP-9* gene is associated with up-regulation of promoter activity (23). In addition, it has been shown that C-1562T *MMP-9* polymorphism influence on gene expression of *MMP-9* (13). These results suggest that the synergism of genotypes and alleles of G-1575A *MMP-2* and C-1562T *MMP-9* gene polymorphisms can have an impact on male fertility. 

There are some limitations in this study. The relatively wider range of confidence interval (CI) observed in TT genotypes of C-1562T *MMP-9* gene polymorphism (0.52-3.98) was probably due to the lower sample size requited in this study. Further studies should be performed with larger sample size together with the determination of MMP-9 and MMP-2 activities to provide more information about the impact of these polymorphisms on male infertility. 

## Conclusion

Our results suggest that the T allele carriers of C-1562T *MMP-9* gene polymorphism have lower immotile sperm number. In addition, a relationship was observed between combined *MMP-2* and *MMP-9* variant genotypes and male infertility. Therefore, it can be concluded that genetic polymorphisms in matrix metalloproteinases may impact on male fertility. 
